# Secretion of Epstein-Barr Virus-encoded BARF1 oncoprotein from latently infected B cells

**DOI:** 10.1186/1743-422X-5-70

**Published:** 2008-06-04

**Authors:** Sylvie Fiorini, Tadamasa Ooka

**Affiliations:** 1 Laboratoire de Virologie Moléculaire, Virologie et Pathogenèse Humaine, FRE 3011, CNRS, Faculté de Médecine Laennec, Université Claude Bernard Lyon-1, Rue Guillaume Paradin, 69372, Lyon Cedex 08, France

## Abstract

Epstein-Barr virus (EBV) encodes two oncogenes, LMP1(Latent Membrane Protein-1) and BARF1 (BamH1-A Reading Frame-1). LMP1 belongs to latent gene family and BARF1 is considered so far as one of early gene family. However BARF1 oncogene was expressed highly in Nasopharyngeal (NPC) and gastric (GC) carcinoma as a type II latency, and in EBV-positive Akata cell and primary epithelial cell infected *in vitro *by EBV as type I latency. Its expression was also reported in Burkitt's lymphoma's biopsy frequent in Malawi in Africa as well as in nasal NK/T-cell lymphoma. We recently observed a massive secretion of BARF1 protein in serum and saliva of NPC patients. NPC-derived c666-1 epithelial cells also expressed and secreted BARF1 protein without other lytic genes expression. We asked whether this oncogene belongs to latent gene family. To investigate, we examined its transcriptional and translational expression in IB4 and Akata B cells where both cell lines belong to latent cell family. Transcriptional expression was analyzed by RT-PCR. As BARF1 protein is one of secreted proteins, its translational expression was analyzed by immunoblot after concentration of culture medium. Secreted BARF1 protein was futher purified by concanavalin A affinity column. BARF1 was transcribed in both EBV-positive AKATA and IB4 cells, and BARF1 protein was secreted from these latently infected human B cells. Its secretion does not depend EBV genome form in infected cells. Both episomal and integrated form of EBV genome were capable of expressing BARF1 gene. These results suggests that BARF1 is expressed in latent stage and increases its expression during lytic stage.

## Background

Epstein-Barr virus (EBV) is tightly associated with divers human cancers, in particular nasopharyngeal and gastric carcinomas, lymphoma induced in Aids patient, Hodgkin's lymphoma and endemic Burkitt's lymphoma [1]. EBV immortalises primary simian and human B-lymphocytes [1,2] as well as epithelial cells *in vitro *[3,4]. EBV infection is latent in B cells and classified in three types: Type I, Type II and Type III. Type I is common to lymphomas and express very limited viral protein, mainly EBNA1, EBERs and BARF0. Type II express EBNA1, LMP1, EBERS, BARF0 and LMP2. Type III express several viral proteins like EBV-encoded nuclear antigens (EBNA1, EBNA2, EBNA3A, 3B and 3C), LMP1, LMP2A, LMP2B, BARF0 and EBERs [5]. Nasopharyngeal carcinoma belongs to Type II. Primary epithelial cells immortalized *in vitro *by EBV expressed EBNA1, EBERs, LMP2A and BARF1[3,4,6], thus belong to type II infection except for the absence of LMP1 expression.

Among about 90 genes encoded by EBV genome, two oncogenes, LMP1 and BARF1, are known to induce a malignant transformation in established rodent fibroblasts [7,8]. BARF1 was considered so far as an early gene. However among the viral lytic proteins, only BARF1 was expressed consistently and at high levels in NPC [9-11] and also in EBV-associated GC carcinoma as well as in EBV-immortalized epithelial cells *in vitro *[3,6,12]. In these cells, the expression of LMP1 and lytic genes was negative. BARF1 expression was also detected in B lymphoma frequent in Malawi [13] and in nasal NK/T-cell lymphoma [14]. Its expression is therefore not limited to epithelial cells, but also in B cells.

BARF1 has a malignant transforming activity in rodent fibroblasts and in human EBV-negative B cells [8,15]. Its transforming and Bcl2 activating domain was demonstrated between 21^st ^to 56^th ^amino acid sequences by deletion mutants [15]. BARF1 has also immortalizing activity on primary primate epithelial cells [16]. Secreted BARF1 protein (called p29) purified from 293 cells infected BARF1 recombinant adenovirus showed hexamer oligomeric structure determined by crystallography analysis [17] and p29 acts as a powerful mitogene [18] under this form. Glycosylation and phospholylation is an important step to become biologically functional [19,20]. This oncoprotein is massively secreted in the serum of NPC patients. Purified BARF1 from serum showed a powerful mitogenic activity [21]. The p29 protein can complex *in vitro *with CSF1 (Colony Stimulating Factor-1) and result in the inhibition of macrophage activation [22] and can also inhibit the secretion of INF-alpha [23]. BARF1 was also recognized by NK cells in ADCC (Antibody-dependent cellular cytotoxity) test [24]. BARF1 is therefore involved not only in oncogenic mechanism, but also in immunomodulation.

As BARF1 was expressed in type II latency and in EBV-immortalized epithelial cells as well as in gastric carcinoma where LMP1 and lytic genes expression were totally absent, our question was addressed whether BARF1 gene belongs to latent gene family. We therefore examined its transcriptional and translational expression in latently infected IB4 (two copies of integrated EBV genome per cell) and type I-AKATA (circular episomal form) cells. This study will permit also to analyse whether integrated EBV genome is capable of producing BARF1. At translational level, we examined secretion of p29 BARF1 protein in cell culture.

## Findings

**Figure 1:**
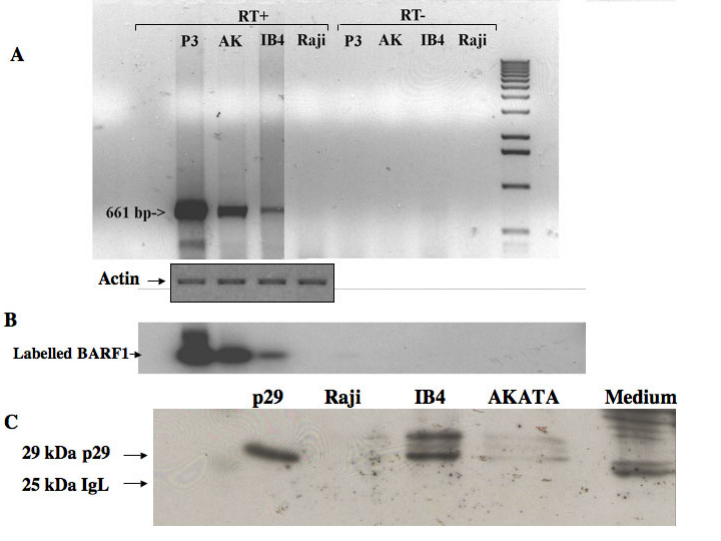
**Transcriptional and translational expression of BARF1 in latently infected AKATA and IB4 cells**. **A. Transcriptional expression of BARF1 on EBV-positive cell lines and BARF1 negative Raji cell line by RT-PCR**. mRNA was purified using bead polyA extraction column (Promega, France). Five μg of mRNA was used for first-strand cDNA synthesis using oligo(dT)_15 _as primer. Reverse transcription was done with Superscript reverse transcriptase (GIBCO, BRL). Amplifications of cDNA were performed in a DNA thermal cycler using the previously described primers (12). Amplified fragment was electrophoresed on 2% agarose gel, then transfered onto nitrocellulose. RT+: with reverse transcriptase. RT-: PCR directly with RNA without reverse transcription. Amplified actin sequence was presented as Actin. **B. Radioactive hybridization**. The hybridization was carried out in 6 × SSC, 0.5% SDS, 3 × Denhart and 200 μg/ml denatured salmon sperm DNA [8], with 10^6 ^cpm/ml of labelled probe [25]. The filter was exposed for 2 hours at -80°C, then developed. **C. Presence of p29 BARF1 protein in culture medium of EBV-positive cell line (IB4 and AKATA) and BARF1-negative Raji cell line**. To purify secreted BARF1 protein, the concentrated medium was incubated with concanavalin A-ag at room temperature, then concanavalin A-ag was washed and elution of the conA-bound proteins was carried out by competition with methyl-ζ-D-glucopyranocide (MGP, 0.5–1.0 M; Sigma) as already described (20). 29 kDa corresponds to M.W of purified BARF1 protein. 25 kDa cprresponds to light chain of immunoglobuline.

We first examined whether BARF1 gene is transcribed in latent stage of EBV analyzing its expression in IB4 and EBV-positive AKATA cell lines. Raji cell line which is EBV genome positive, but defective to BARF1 sequence was used as a negative control and P3HR-1 cell line as a positive control. For detection of its transcript, we used RT-PCR using primers 5'-GGGGATCCCAGAGCAATGGCCAGGTTC-3' as anti-sens BARF1 sequence and 5'-GGGGATCCAAGGTGAAATAGGCAAGTGCG-3' as sens BARF1 sequence, giving 661 bp [10]. For actin, primers were used 5'-CCTTCCTGGGCATGGAGTCCT-3' (sens) and 5'-GGAGCAATGATCTTGATCTTC-3' (anti-sens). The cDNA sequence was amplified by PCR, and amplified fragment was first analyzed by UV light, then confirmed by specific radioactive hybridization method. As illustrated in figure 1, amplified BARF1 sequence was detected in P3HR-1 and absent in negative control Raji (Fig. 1A). EBV-positive AKATA and IB4 cell lines gave a positive response for BARF1 transcription. Positive sequences were found only in RT^+^, but not in RT^- ^(direct amplification of mRNA)(Fig. 1A), suggesting that positive response came from BARF1 mRNA and not from contaminating DNA sequence. As previously described [25], an entire *BARF1 *sequence was detected by hybridization using a ^32^P-labelled *BARF1 *probe prepared with a random-primer DNA-labeling. Hybridization experiment confirmed that the amplified fragments visualized in figure 1A were specific BARF1 sequence (Fig. 1B). In comparison with actin expression, P3HR1 transcribed BARF1 mRNA much higher than EBV-positive AKATA cells. Lower expression of BARF1 in IB4 comes probably from its low EBV copy number (two genome copies per cell).

In second, translational expression of BARF1 gene in latent stage of EBV was examined in IB4, EBV-positive AKATA and Raji cell lines. The p29 purified from 293 cells infected with BARF1-recombinant adenovirus was used as a positive control. At translational level, this oncoprotein was difficult to be detected in cellular extract from EBV-positive cells, because almost all p29 was secreted outside of cells. This rendered so far difficult to evaluate its expression in cells expressing latent and lytic phase. In fact, when we analysed cellular extract from AKATA and IB4, we could not identified BARF1 protein (data not-shown). We therefore analysed the presence of secreted BARF1 protein in cuture medium. As previously described by Sall *et al. *[18], secreted BARF1 protein was prepared from 10 liters of AKATA, IB4 and Raji cell culture. Culture medium was finally concentrated to 4 ml (resulting 2500 folds concentration). As BARF1 protein has affinity for agarose-conjugated concanavalin A [20,21], concentrated culture medium was purified with Concanavalin A. Affinity purified BARF1 protein was analyzed on 12% polyacrylamide gel. Expression of BARF1 was detected by polyclonal antibody PepIII (produced by rabbit injected with peptide NGGVMKEKD corresponding to aminoacids 172 to 180) [10] by using an enhanced chemiluminescence system. We could detect p29 protein in concentrated medium from EBV-positive AKATA and IB4 cells, while such band was never detected in concentrated Raji medium as well as concentrated RPMI medium containing 10% FCS (Fig. 1C). IB4 cells secreted much higher p29 than AKATA cells. This is contrarly to their transcription, although BARF1 quantity could not be quantified by actin standard marker due to their secreted protein statue.

We demonstrated in this study the expression of BARF1 in type I AKATA and latently infected IB4 cells at transcriptional and translational level. Our recent data showed that the BARF1 p29 protein was massively secreted in serum from NPC patients [21]. BARF1 was also secreted in culture medium of NPC-derived c666-1 epithelial cells [21] in where no translational expression of any lytic gene was detected [26]. Its expression was recently demonstrated in B-lymphoma frequent in Malawi [13] and in nasal NK/T-cell lymphoma [14]. B lymphoma developed in Tamarin after injection of EBV also expressed BARF1 [27], while no lytic genes were expressed [27]. Taking together, BARF1 was expressed in letently infected cells and not limited to epithelial cells, but also in B cells in which there are no expression of any lytic genes. We also showed in this report that BARF1 protein was translated from both integrated and epsomal EBV genome. From our two recent observations, 1) a powerful mitogenic activity of BARF1 purified from serum of NPC patient [21] and 2) BARF1 protein purified from BARF1-recombinant adenovirus-infected 293 cells possess also a powerful mitogenic activity on human Louckes B cells [18], secreted BARF1 protein from B and epithelial cells has an important role in immunoregulation [22-24] and/or in activation of cell cycle during tumor development [21].

## Competing interests

The authors declare that they have no competing interests.

## Authors' contributions

SF contributed to perform the experiment. TO contributed to design, also perform the experiment and draft the manuscript. All authors read and approved the final manuscript.
